# Prevalence and determinants of violence against health care in the metropolitan city of Peshawar: a cross sectional study

**DOI:** 10.1186/s12889-021-10243-8

**Published:** 2021-02-10

**Authors:** Muhammad Naseem Khan, Zia Ul Haq, Mirwais Khan, Sadia Wali, Faryal Baddia, Shaista Rasul, Salman Khan, Maciej Polkowski, Jessica Yohana Ramirez-Mendoza

**Affiliations:** 1 Khyber Medical University, Institute of Public Health & Social Sciences (IPH&SS), Phase V, Hayatabad, Peshawar, Pakistan; 2 Department of Psychological Medicine, University of Liverpool, Liverpool, UK; 3 International Committee of the Red Cross, Geneva, Switzerland

**Keywords:** Healthcare personnel, Physical violence, Verbal violence, Pakistan, Peshawar

## Abstract

**Background:**

Violence against healthcare personnel is a major public health problem. Healthcare personnel are at the frontline dealing with people in stressful and unpredictable situations. Therefore, this study was conducted to determine the prevalence and associated factors of violence against health care personnel.

**Methods:**

A cross sectional study was conducted in the district Peshawar. Healthcare personnel from public and private sectors working in both the primary and tertiary levels of healthcare were invited to participate. Violence was assessed through a structured questionnaire previously used in Pakistan and was defined as experiencing and/or witnessing any form of violence in the last 12 months. Mental health was assessed through the General Health Questionnaire. Logistic regression was used to estimate the association of violence against healthcare personnel with psychological distress and demographic characteristics. Data entry and analysis were conducted in STATA 14.

**Results:**

A total of 842 healthcare personnel participated in the study. The prevalence of violence experienced and/or witnessed by healthcare personnel in Peshawar was 51%. Verbal violence remained the predominant form of violence and almost half of the healthcare personnel (45%) were exposed to it. A quarter of the respondents (24%) reported physical violence alone or in combination with other forms of violence. In almost two third of the incidents the perpetrators were either attendants, relatives or the patients. The emergency unit and wards within healthcare facilities were the most common places where violent events took place. The major factors responsible for the violent incidents were communication failure, unreasonable expectations and perceived substandard care. No uniform policy/procedure existed to manage the incidents and the healthcare personnel adopted different responses in the wake of violent events targeting health care. Working in public healthcare facilities and having a larger number of co-workers/colleagues significantly increased the risk of violence in the healthcare settings while being a paramedic significantly reduced the risk as compared to physicians.

**Conclusions:**

Violence against healthcare personnel is a serious public health issue and the prevalence is quite high. A holistic effort is needed by all stakeholders including healthcare community, the administration, lawmakers, law enforcement, civil society, and international organizations.

## Background

The World Health Organization (WHO) defines violence as “the intentional use of physical force or power, threatened or actual, against oneself, another person, or against a group or community that either results in or has a high likelihood of resulting in injury, death, psychological harm, mal-development, or deprivation” [1, 2]. Similarly the World Health Organization defines workplace violence as “incidents where staff are abused, threatened or assaulted in circumstances related to their work, including commuting to and from work, involving an explicit or implicit challenge to their safety, well-being or health” [3]. Healthcare personnel are ranked as one of the most exposed group experiencing violence and aggressive behavior. According to the International Labor Office (ILO), workplace violence affects all of the sectors and workers, however health sector is the most highly affected sector [4]. Therefore, violence against healthcare personnel is a major public health problem. Workplace violence can be physical, sexual or psychological in nature and can be actual or threatened [3].

Violence against healthcare personnel is a global phenomenon and studies have been conducted to report the prevalence and associated factors. Studies have reported violence in the Western World [5, 6], Middle East [7] and sub-Saharan Africa [8]. In Pakistan, violence against healthcare personnel is not a new area and has been studied previously. A nationwide study in the major tertiary care hospitals of Pakistan reported more than 70% healthcare personnel in the emergency departments having experienced some form of violence in the 2 months preceding the study. While physical abuse was faced by 12% physicians, verbal abuse was experienced by 65% [9]. The study found that male physicians were more likely to be victims of violence. Similar findings were reported by another study in Karachi, where one in six and three in five physicians reported physical or verbal abuse, respectively, in the past 12 months [10]. In another research study on violence against healthcare almost two thirds of the participants had either experienced or witnessed some kind of violence in the 1 year preceding the study. The main reasons for violence included unreasonable expectations, communication failure, human error, unexpected outcomes, and perception of substandard care [11]. Likewise, emergency department and wards were the most common sites of violence. Due to the front line nature of the work, violence in the emergency department is more prevalent than in other areas of healthcare facilities [12]. Lack of preventive policies, educational inadequacy, unwillingness to report assaults given that violence is considered as part of the job by the healthcare personnel, and unmet expectations of patients and their family are some of the major reasons for violence [13].

Healthcare personnel face particular risks as they are at the frontline dealing with people in stressful, unpredictable and potentially volatile situations. Research on violence against healthcare has been conducted mostly in the southern part of the country mainly Karachi, with no local research on the prevalence, types, and major reasons of violence against healthcare personnel in district Peshawar. Peshawar is located in the northern part of the country with different language, socioeconomic levels, ethnicities, values and culture; therefore, the objectives of the current study were to determine the prevelance and associated factors of violence against healthcare in district Peshawar and also find out association of this violence with the psychological health of the healthcare personnel. The current study was conducted to address this knowledge gap to enable policy makers and planners to develop evidence-based measures for the prevention and control of violence against healthcare.

## Methods

### Study design and settings

A cross-sectional study design was employed for this research. The study was conducted in district Peshawar from April to November of 2017. Healthcare personnel including physicians, nurses, paramedical staff and supporting staff (ward orderlies, ambulance drivers, and gate keepers) were included in the study. The participants were invited from the three main public sector tertiary care hospitals (total 3 in number in district Peshawar) and a sample of private tertiary care hospitals and primary healthcare facilities. Nine Basic Health Units (BHUs) and three Rural Health Centers (RHCs) were randomly selected from the list of primary healthcare facilities in the district. Similarly from the list of private sector tertiary care hospitals two were randomly selected.

Sample size calculations were based on the anticipated frequencies of verbal and physical abuse taken from a previous study conducted in Karachi, Pakistan [11]. The sample size was calculated for each of the main strata based on the assumptions of an alpha of 0.05, confidence interval of 95% and a prevalence of violence from a previous study in Karachi of 65.9%. The sample size based on these assumptions and the actual number in each category was calculated through OpenEpi version 3.01 [14]. The total sample size for physicians was 293, nurses 284, paramedics 274 and support staff 270. Assuming a non-response rate of 10%, additional sample was enrolled within each of the four main categories making a total sample size of 1233. The final sample was proportionally invited from each of the main tertiary care hospitals.

A multistage sampling technique was used. Human resources data from the three-major tertiary care public hospitals was provided by the concerned administration entities and was grouped as physicians, nurses, paramedical staff and support staff. Further stratification was done by segregating the above categories into those working in other departments and those specifically deployed in the Accident and Emergency department. Further stratification was done based on their level of seniority as follows: physicians were stratified into senior physicians (registrar and above) and junior physicians (trainee physicians, medical officers and house officers); nurses were stratified into head nurse and staff nurse; paramedics into senior technicians and junior technicians; and support staff into ambulance drivers, computer operators, ward orderlies and security personnel. For private hospitals and primary healthcare centers, cluster sampling technique was applied. Therefore, at first, a random cluster of centers were selected from all the public and private sector hospitals. In the second stage all the healthcare personnel working in CDs/BHUs were sampled for the study while for RHCs and private hospitals, 50% of physicians, nurses, paramedics and support staff were randomly selected.

Healthcare personnel with at least 1 year of work experience in the healthcare settings were enrolled in the study. Healthcare personnel with less than a year of professional experience, those who were on sick/maternity leave, and those who retired during the study period or were transferred at the time of data collection were excluded.

### Measures

#### Violence against healthcare personnel

Violence was assessed through a modified instrument from the *Joint Programme on Workplace Violence in the Health Sector* of the International Labour Office, the International Council of Nurses, the World Health Organization, and the Public Services International [15]. This instrument has been translated to the local language (Urdu) and used in similar studies conducted in Karachi [10, 11]. For this study, violence was defined as any individual or group aggressive behavior or exercise of power, which is socially non-acceptable, turbulent, and often destructive. It was mainly assessed whether the respondent had experienced or witnessed any form of violence in the last 12 months. Physical violence was defined as the use of “physical force against another person that results in physical, sexual, or psychological harm and includes beating, kicking, slapping, stabbing, shooting, pushing, biting and pinching, among others”. Verbal abuse included “bullying, mobbing, harassment, and verbal abuse that humiliates, degrades or otherwise indicates a lack of respect for the dignity and worth of an individual”. Information gathered from the participants of the study included demographics of the healthcare personnel and administrative information of the healthcare institution. Specific workplace violence questions allowed to assess how worried the healthcare personnel was regarding violence in the healthcare settings; which was rated on a scale from *not at all* to *very worried*. Furthermore, details on specific incidents of violence were collected to assess whether violence was physical or verbal, the incident frequency, the type of perpetrators, the location where violence took place within the healthcare institution, and the time of the incident. Healthcare personnel provided information about the main reasons behind the incident, the response following the incident of violence by the healthcare personnel and their recommendations to reduce such incidents or mitigate the impact of such incidents in the future.

#### Psychological distress

Psychological distress was measured through the General Health Questionnaire (GHQ-12) [16]. It’s score ranges from 0 to 36 and were defined as follows: score < 12 were coded as normal, 12–20 as distress and more than 20 as severe distress. GHQ-12 has been used and validated in Pakistan with around 90% sensitivity and specificity [17].

### Data collection and analysis

Before actual data collection, a team of research assistants with experience in data collection were trained on the data collection instruments. Data were collected daily from the research assistants at KMU by the research coordinator. The research coordinator handed over the data files to data entry team for entry/cleaning of data. Any discrepancy and missing data were reported back immediately to the research assistants for clarification. Data entry was done in STATA 14. Mean and standard deviation measures was used for descriptive statistics of scale data, and frequencies and percentages for categorical data. For association of violence against healthcare personnel and psychological distress and other demographic factors chi square was used for uni-variate analysis and logistic regression for multivariate analysis. Associations were considered significant at *p* value of < 0.05.

#### Ethical approval

The research proposal was reviewed by the institutional and university level boards and committees, along with the approval from the hospital administration. Ethical approval was obtained from the Ethics Board of Khyber Medical University, Peshawar. Informed written consent was obtained from every participant after explaining the purpose of research and providing written information to participants. The right to withdraw from the study at any time without providing a reason was reinforced to all participants during consent and prior to the conduct of the survey.

## Results

A total of 1283 healthcare personnel were approached for participation in the current study. Of them 187 were part of the exclusion criteria of having less than 1 year of professional experience, were transferred, retired or on leave and as such were excluded from the study. An additional 204 participants refused participation. Lastly, 50 forms were incompletely filled and hence were excluded from analysis. Therefore, analysis was done on a total of 842 healthcare workers.

### Demographic and job characteristics

Table 1 below shows the demographic and job characteristics of the participants and their associations with exposure to violence in the last 1 year. Statistically significant associations were observed between violence and number of co-workers, public healthcare facility, and job categories of the healthcare personnel. Healthcare personnel exposed to violence had significantly higher levels of preoccupation about workplace violence with 24.6% of them feeling worried and 17.8% extremely worried; while among the healthcare personnel not exposed to violence, only 2.7% felt worried and 2.9% extremely worried. The current study found that age, work experience, gender, marital and distress status of healthcare personnel, and level of the healthcare facility (primary or tertiary) had no association with exposure to violence.

**Table 1:** Demographic & Job Characteristics and association with Violence

Variables	Exposed to violence *N* = 427 n (%)	Not exposed to violence *N* = 415 n (%)	Significance
Age in years (Mean ± SD)	35.44 ± 9.2	35.52 ± 10.0	0.910
Work Experience in years (Mean ± SD)	9.85 ± 8.88	9.85 ± 8.67	0.868
No of co-workers (Mean ± SD)	7.14 ± 7.22	4.90 ± 4.68	< 0.001
**Gender**			
Male	296 (69.32)	278 (66.99)	0.468
Female	131 (30.68)	137 (33.01)	
**Level of healthcare facility**			
Primary	37 (8.7)	38 (9.2)	0.802
Tertiary	390 (91.3)	377 (90.8)	
**Type of Health facility**			
Public	396 (92.7)	298 (71.8)	< 0.001
Private	31 (7.3)	117 (28.2)	
**Marital status**			
Single	116 (27.2)	123 (29.6)	0.426
Married	311 (72.8)	292 (70.4)	
**Job category**			
Physicians	104 (24.4)	68 (16.4)	< 0.001
Nurse	84 (19.7)	109 (26.3)	
Paramedics	89 (20.8)	126 (30.4)	
Support Staff	150 (35.1)	112 (27.0)	
**How worried are you regarding violence in your work settings**			
Not worried	106 (24.8)	226 (54.5)	< 0.001
Somewhat worried	57 (13.4)	92 (22.2)	
Little worried	83 (19.4)	74 (17.8)	
Worried	105 (24.6)	11 (2.7)	
Extremely worried	76 (17.8)	12 (2.9)	
**Distress status**			
Normal	206 (48.2)	214 (51.6)	0.621
Somewhat distress	200 (46.8)	181 (43.6)	
Severe distress	21 (5.0)	20 (4.8)	

### Prevalence & pattern of violence

The prevalence and patterns of different types of violence against health care (verbal, physical and damage to facility) is presented in Table 2. There was exposure to violence among 51% of the sampled healthcare workers in Peshawar district over a period of 1 year. More than a quarter of the respondents (26%) had experienced as well as witnessed violence against health care. Among respondents who had witnessed and/or experienced violence, verbal violence remained the most prevalent form (45%), followed by a combination of physical and verbal abuse (23%) and a combination of physical, verbal and facility damage (22%). A higher prevalence of violence was observed in public healthcare facilities, and among physicians and support staff compared to nurses and paramedics.

**Table 2:** Prevalence & Pattern of violence

Prevalence of Violence	***N*** = 427 n (%)
Experienced and/or Witnessed	427 (51)
Experienced only	33 (4)
Witnessed only	176 (21)
Experienced and Witnessed	218 (26)
**Type of healthcare facility**	
Public	396 (93)
Private	31 (7)
**Level of healthcare facility**	
Primary healthcare facility	37 (9)
Tertiary healthcare facility	390 (91)
**HealthCare Personnel**	
Physicians	104 (24)
Nurses	84 (20)
Paramedics	89 (21)
Support Staff	150 (35)
**Gender of the healthcare personnel**	
Male	296 (69)
Female	131 (31)
**Pattern of violence**	
Verbal violence only	192 (45)
Verbal & Physical violence	100 (23)
Verbal, Facility damage & Physical violence	92 (22)
Only Physical violence	3 (0.7)
Only Facility damage	2 (0.6)
Physical & Facility damage	3 (0.7)
Verbal & Facility damage	35 (8)

Table 3 shows the characteristics of the violent incidents. In more than two thirds of the incidents the perpetrator was either the attendant/relative of the patient (44%) or the patient (20%). In 85% of incidents there was involvement of two or more perpetrators in each event. Emergency departments (34%) and wards (30%) were the common sites of violent incidents in the healthcare facilities, and more than half (52%) of the incidents took place during the morning shift.

**Table 3:** Characteristics of the violent incidents

Characteristics of violent incidents	Percentages
**Perpetrators of violence**	
Attendant/relative	44
Patient	20
General public	14
VIP escort	8
Staff member	7
Security personnel	5
Others	2
**Number of perpetrators involved**	
One	15
Two-five	72
More than five	13
**Number of violent incidents experienced and/or witnessed over 12 months**	
Once	12
Two-five times	55
More than five	33
**Number of victims affected in violent incidents**	
One	40
Two-five	55
More than five	5
**Place of violence within healthcare facility**	
Emergency room	34
Ward	30
Hospital parking area	8
Radiology	8
Intensive Care Unit	5
Others	15
**Distribution of the violent events by time**	
Morning shift (8 to 2)	52
Evening (2–8)	20
Night (8)	13
Do not remember	15
**Consequences of violence**	
Injured victims requiring treatments	75

### Mental health effects of violence

**Fig. 1:**
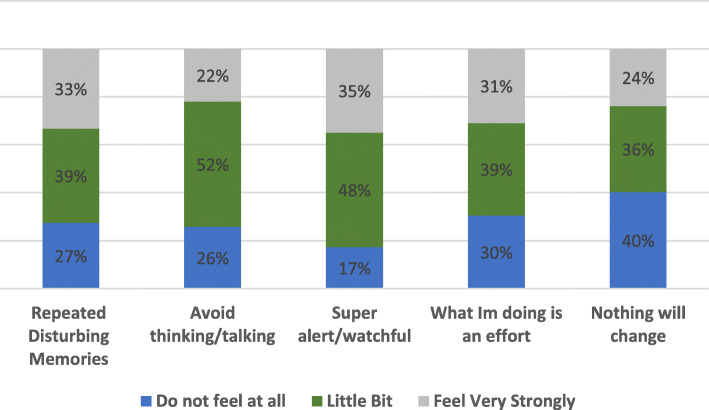
Mental health effects of the violence incident

The mental health effects on healthcare personnel who had witnessed and /or experienced violence are summarized in Fig. 1 below. Around two-thirds of the participants exposed to violence had experienced some form of mental health consequences following the incidents. Regarding the GHQ 12 status, half of the respondents had a score of less than 12, 45% were distressed with a score of 12–20, while the remaining 5% were severely distressed with a score of more than 20.

### Factors responsible and events following the violent incidents

Table 4 summarizes the factors responsible for the development of violence against healthcare and the actions taken by the victims following the events. The table also shows the factors that the respondents of the study deemed as generally contributing to the development of violence against healthcare and the recommendations made by the participants for the prevention of violence in healthcare settings. Communication failure (71%), unreasonable expectations (61%), perceived substandard care (55%), management failure (55%) and human error (51%) were the predominant factors responsible for the violent incidents. Other important factors included financial pressure, facility failure, a smaller number of staff and unexpected outcomes. Following the incident around two thirds (61%) defended themselves and around half (52%) told the attacker to stop, the victim sought counselling (43%) and tried to report the incident to police (45%). The healthcare personnel were asked to identify factors generally contributing to violence in healthcare settings. The respondents identified low awareness among the general public, workload of healthcare personnel, lack of facilities, shortage of staff and high expectations as major factors contributing to violence against health care. The participants reported improvement in healthcare services, increase of healthcare personnel, improvement in overall public awareness, controlled entry of attendants, provision of security and abolition of VIP protocols as recommendations for the prevention of violence in the healthcare settings.

**Table 4:** Factors responsible and events following the violent incidents

Characteristics of the violence incident	n (%)
**Factors responsible for the violent incidents (multiple responses)**	
Communication failure	302 (71)
Unreasonable expectation	261 (61)
Management failure	234 (55)
Perceived substandard care	233 (55)
Human error	219 (51)
Financial pressure	195 (46)
Less number of healthcare personnel	147 (34)
Unexpected outcome	128 (30)
**Responses of the victims following the violent incident (multiple responses)**	
Tried to pretend it never happened	136 (32)
Attacker was told to stop	222 (52)
The victim defended him/herself	260 (61)
Sought counseling	182 (43)
Sought help from association	194 (45)
Reported to Police	194 (45)
**Reasons for not taking any action (multiple responses)**	
It was not important	156 (66)
Felt ashamed	52 (22)
Felt guilty	33 (14)
Useless	98 (41)
Afraid of negative consequences	80 (34)
Did not know who to report to	66 (28)
**Factors contributing to violence in health-care settings (multiple responses)**	
Low awareness in the general public	488 (58)
Workload of healthcare personnel	429 (51)
High expectations	224 (27)
VIP protocols	138 (16)
Lack of facilities at the healthcare facility	290 (34)
Shortage of healthcare personnel	245 (29)
**Recommendations for the prevention of violence (multiple responses)**	
Improvement in healthcare services	585 (70)
Healthcare personnel increase	447 (53)
Provision of security	219 (26)
No VIP protocols	129 (15)
Controlled entry of attendants	289 (34)
Overall public awareness	390 (44)

Table 5 shows the associations of violence against healthcare personnel through regression analysis with presentation of adjusted and unadjusted odds ratios with 95% confidence intervals. The likelihood of experiencing and/or witnessing violence was significantly associated with public healthcare facilities, larger number of co- workers/colleagues, worry regarding violence in the healthcare settings and job category. Working in public healthcare facilities, worry regarding violence in the healthcare settings and having a larger number of co-workers/colleagues significantly increased the odds of a healthcare personnel experiencing and/or witnessing violence while being a paramedic significantly reduced the odds as compared to physicians.

**Table 5:** Adjusted and unadjusted Odds ratio of Violence in the healthcare settings

Variables	Unadjusted Odds ratio (95% CI)	P value	Adjusted Odds ratio (95% CI)	P value
Age in years	0.10 (0.99, 1.01)	0.910	0.98 (0.96, 1.01)	0.256
Work Experience in years	1.00 (0.99, 1.02)	0.868	1.00 (0.96, 1.03)	0.826
No of co-workers	1.07 (1.04, 1.09)	< 0.001	**1.06 (1.03, 1.09)**	**< 0.001**
Male	1.11 (0.83, 1.49)	0.468	1.03 (0.64, 1.64)	0.903
Tertiary healthcare facility	1.06 (0.66, 1.71)	0.802	0.71 (0.40, 1.25)	0.233
Public sector health facilities	5.02 (3.28, 7.66)	< 0.001	**3.82 (2.35, 6.19)**	**< 0.001**
Married	1.13 (0.84, 1.52)	0.426	1.15 (0.76, 1.73)	0.503
**Job category**				
Physicians	Reference category			
Nurse	0.50 (0.33, 0.77)	0.001	0.82 (0.45, 1.50)	0.518
Paramedics	0.46 (0.31, 0.70)	< 0.001	**0.59 (0.36, 0.97)**	**0.038**
Support Staff	0.88 (0.59, 1.30)	0.006	1.55 (0.90, 2.68)	0.115
**How worried are you regarding violence in your work setting**				
Not worried	Reference category			
Somewhat worried	1.32 (0.88, 1.98)	0.176	1.39 (0.89, 2.17)	0.149
Little worried	2.39 (1.62, 3.53)	< 0.001	**2.85 (1.83, 4.42)**	**< 0.001**
Worried	20.35 (10.49, 39.48)	< 0.001	**17.91 (8.94, 35.87)**	**< 0.001**
Extremely worried	13.50 (7.04, 25.89)	< 0.001	**14.10 (7.10, 28.02)**	**< 0.001**
**Distress status**				
Normal	Reference category			
Somewhat distress	1.15 (0.87, 1.52)	0.330	0.70 (0.48, 1.00)	0.051
Severe distress	1.09 (0.57, 2.07)	0.791	0.57 (0.25, 1.28)	0.174

## Discussion

The current study was the first of its kind to determine the prevalence of violence against healthcare personnel and the associated factors through a large representative sample of both the public and private healthcare sectors, including primary and tertiary levels of care, in district Peshawar, Pakistan. The prevalence of violence witnessed and/or experienced by healthcare personnel in Peshawar was 51%. Verbal violence remained the predominant form of violence witnessed and/or experienced. Almost half of the healthcare personnel (45%) involved in the study had witnessed and/or experienced verbal violence. Furthermore, a quarter of the respondents (24%) reported witnessing and/or experiencing physical violence alone or in combination with other forms of violence. In almost two third of the incidents the perpetrators were either attendants, relatives of patients or the patients. The emergency unit and wards within healthcare facilities were the most common places where violent events took place. The major factors responsible for the violent incidents were communication failure, unreasonable expectations and perceived substandard care. No uniform policy/procedure existed to manage the incidents and the healthcare personnel adopted different responses in the wake of violent events targeting healthcare. Working in public healthcare facilities and having a larger number of co-workers/colleagues significantly increased the risk of violence in the healthcare settings while being a paramedic significantly reduced the risk as compared to physicians.

The main strength of the current study was that the sample was drawn from the official records and healthcare workers were invited randomly, reflecting the true prevalence of violence against healthcare. Furthermore, healthcare workers were invited from public/private and primary/tertiary level of care. The main limitation of the study was that due to the cross-sectional nature, the temporal relationship between exposure and outcome could not be established. Therefore, a study design exploring the temporal relationship between factors and violence against health care should be employed along with patient/attendant perspective in future studies. Likewise 204 participants refused participation and no baseline information collected to compare with the respondents. This is another limitation as the non-respondents might have been more exposed to violence against healthcare.

Violence against healthcare is not a new phenomenon as healthcare professionals around the world are exposed to some form of violence [1, 9, 10, 18, 19]. The current study portrays a similar picture and reported verbal violence as the most common form of violence compared to physical or other forms [18, 20]. Likewise, physical violence is experienced by (8–38%) of health workers worldwide [1]. The frequency of experiencing/witnessing physical abuse (24%) as per the findings of the study are consistent with this range. However, this figure is considerably higher when compared with the country wise frequency of physical abuse reported by healthcare workers in Bulgaria (7.5%), Brazil (6.4%), Lebanon (5.8%), Thailand (10.5%), South Africa (9–17%) and Karachi, Pakistan (16%) [4, 10, 21]. These different results could be explained by the different recall times in these studies as the shorter period will have better recall, but few incidents compared to the longer period.

The higher rates of physical violence could also be due to the large number of relatives/attendants accompanying the patients in this part of the world and is considered a culutural norm to visit patients even in the hospital. This at time is required to facilitate the management of the patient in the hospital as not all the services and care (laboratory tests/medicines and nursing care) is provided by the hospital staff. In the current study more than one perpetrator was involved in 85% of the incidents of violence. Multiple studies conducted across various cities of Pakistan have revealed that the main perpetrators of violence against healthcare workers were attendants of patients [9, 19]. Therefore, policy should be adopted to limit the entry of relatives/attendants with the patients and raise awareness among the general community on the attendant’s restriction. The shape and impact of implementation of this policy should be guided by continuous evaluation to remain mindful of the cultural needs and the duty to ensure safety of patients and healthcare personnel.

Consistent with the findings of other studies, our results show that amongst the various cadres of healthcare workers, doctors encounter fewer incidents of violence whereas paramedics experienced the greatest number of violent incidents. Communication failure (71%), unreasonable expectations (61%), management failure (55%) and perceived substandard care (55%) were reported as the primary causes of violence against healthcare workers. Communication failure was the biggest reason reported in the current study. This was reported by the healthcare personnel in a study where they believed that their dismissive and authoritative attitude and a lack of empathy contributes to emergence of incidents of violence [22]. The other factors reported have also been reflected in similar studies conducted across various countries including Pakistan [23–25]. There is a lack of a robust referral system in Pakistan. The concept of gatekeeping in healthcare does not exist. Patients tend to seek care at tertiary level facilities even for minor self-limiting ailments. This translates into unrealistic expectations by the patients and puts a toll on the already compromised facilities. Furthermore, it is evident from the findings of this study, the health care workers working in public sector facilities experience violence more frequently as compared to those working in the private sector. Public sector hospitals in Pakistan are overburdened. Healthcare workers working at public hospitals cater to an overwhelmingly large number of patients visiting the facilities at all hours of the day. Under these circumstances the healthcare workers have to rush through the patients and are not being able to give the due time and consideration which every patient deserves, thereby resulting in communication failure, management failure and perceived substandard care. Increasing the capacity of workforce and expansion of the facilities has been proposed by the study participants as one of the strategies to overcome these issues. However, one interesting finding was the association of violence with large number of co-workers/colleagues. This might be because the health workers felt confident with the support and resort to violence easily in such settings. Therefore, expansion of the facilities/recruitment of the staff to deal with the workload should be done with additional training on better communication skills, conflict resolution and de-escalation of the violent events.

Another important factor for violence against healthcare is financial pressure (46%) on part of the patients. This has been reported for violence against healthcare in other low and middle-income countries [26]. In Pakistan 29% of the population lives below the national poverty line. Despite this staggering figure, the out of pocket health expenditure approximates 70% of the country’s net expenditure on health [27]. Recently the government of Khyber Pakhtunkhwa has initiated social health protection schemes (insurance schemes for admitted patients) covering nearly 49 million people living below the poverty line, for all secondary healthcare admission [27, 28]. These schemes could play a big role in reducing the burden of violence against healthcare.

In more than half (61%) of the incidents; the healthcare worker defended himself/herself. Similarly, around half of the healthcare workers verbally told the attacker to stop. One explanation could be that there is no formal system for redressal of these issues at the institutional level. Therefore the healthcare worker has no other choice but to defend him/herself, and the local culture encourages self defense in situation like these. About 45% of the victims sought counselling and/or pursued prosecution whereas 32% pretended that the abuse did not take place at all. The reasons behind the reluctance of healthcare workers in reporting such incidents of violence are manifold. More than half of the healthcare personnel who did not report the incident deemed it not important or futile. Whereas others either felt threatened by the adverse consequences of seeking help and prosecution or felt that reporting such incidents of violence threatened their self-respect and dignity. Therefore, pretending that the incident did not take place at all seemed like the most suitable reaction in such situations for many. These findings are in conformity with similar studies which reported that amongst the reactions to violence against healthcare, feeling angry, helpless and humiliated are the most commonly reported reactions [29, 30]. Why is it that health professionals, who are amongst the most revered members of the society find themselves helpless in such situations? One of the reasons could be a lack of trust in the relevant institutions, in the system of accountability and in the formal process of seeking prosecution which in most cases appears like a time consuming and futile exercise. Another reason, as reported by an Iranian study is the lack of responsibility by the health system for redressal in such cases [31]. One of the major reason for underreporting of incidents of violence by health professionals according to a study conducted in Pakistan was the fear of having adverse effect on the victims job and personal life [32]. Clearly this reflects a lack of trust in the management of incidents of violence by the healthcare organizations. Similarly based upon the factors that lead to incidents of violence, another study conducted in Karachi predicted a 5% reduction in such cases of violence against health professionals by introducing grievance-redressal policies for the staff [33].

Incidents of violence adversely affect the mental health of the healthcare workers. Around half of the healthcare workers (52%) in the study felt mentally distressed due to violence instigated against them. The psychosocial consequences of violence have been widely reported in various studies. A systematic review on the aftermath of violence against healthcare stated that amongst the various consequences of violence the psychosocial affects such as depression and anxiety are experienced most frequently by the victims [34]. This could have major implications where one third of the healthcare personnel avoid and pretend that the incident didn’t took place.

The phenomenon of violence against health care can have devasting public health consequences, especially for fragile, under-resourced healthcare systems. It holds the potential to immensely balloon the healthcare needs, diminish the availability of services and render the access to and provision of healthcare services unsafe and inefficient. For an over-stretched and over-burdened healthcare system, the additional burden imposed by humanitarian consequences of violence against health care can tip the scales between the needs and availability of the healthcare services in a direction detrimental to the health outcomes of populations using the healthcare services.

## Conclusion

Violence against healthcare personnel is a serious public health issue and the prevalence is high. This may have effects on their wellbeing and could lead to low job motivation, which in turn can put the healthcare provision at risk in an already compromised healthcare system in a developing country like Pakistan. Therefore safety of the wounded and the sick, healthcare personnel, healthcare facilities and medical vehicles is important for provision of essential services. Some of the strategies for the prevention and control of violence against healthcare could be

- Development and implementation of “zero tolerance policy for violence in healthcare settings”, filling gaps in legislation and establishment of incident reporting mechanism by the provincial health department, administration of hospitals, and other monitoring bodies.
- The administration of all public and private healthcare facilities should train healthcare workers on de-escalation of violence [35], effective communication and conflict resolution skills, and ethics [36, 37].
- Administration of healthcare facilities, provincial health department and local law enforcement agencies need to implement measures to improve hospital and staff security and strengthen the needed coordination.
- Provincial health department and administration of healthcare facilities should improve facilitation of patients and attendants through clear signposting and information desks, waiting areas, one attendant policy, and improvement of quality of healthcare services.
- Engagement of media, community elders and religious influencers to raise awareness and counter cultural barriers to increased respect for health care.

These should be assessed through research in the local settings with involvement of not just by the healthcare community, but also needs the support and facilitation from government, the administration, lawmakers, law enforcement, civil society, and international organizations.

## Data Availability

The raw data available upon reasonable request from the corresponding author.

## Abbreviations

BHU: Basic Health Units
CD: Civil Dispensary
HCiD: Health Care in Danger
GHQ: General Health Questionnaire
ICRC: International Committee of the Red Cross
ILO: International Labor Office
KMU: Khyber Medical University
RHC: Rural Health Center
WHO: World Health Organization
